# The epidemiology of herpes zoster and its complications in Medicare cancer patients

**DOI:** 10.1186/s12879-015-0810-6

**Published:** 2015-02-27

**Authors:** Mihran A Yenikomshian, Adrienne P Guignard, François Haguinet, Ann S LaCasce, Arthur T Skarin, Alex Trahey, Paul Karner, Mei Sheng Duh

**Affiliations:** Analysis Group, Inc., 111 Huntington Ave., Tenth Floor, Boston, MA 02199 USA; GlaxoSmithKline Vaccines, Wavre, Belgium; Dana-Farber Cancer Institute, Boston, MA USA

**Keywords:** herpes zoster, Neoplasms, Incidence, Elderly, Risk factors, Neuralgia, Postherpetic neuralgia

## Abstract

**Background:**

Literature on the epidemiology of herpes zoster (HZ) in cancer patients is sparse and does not include the elderly. The objectives of this study were to determine the incidence of HZ and related complications in elderly cancer patients and assess risk factors associated with HZ.

**Methods:**

Patients ≥65 years diagnosed with cancer in 1991–2007 were identified from the Surveillance, Epidemiology, and End Results (SEER) cancer registry-Medicare linked database in this retrospective, longitudinal, open cohort study. The observation period spanned from first cancer diagnosis until the end of data availability. A random group of non-cancer Medicare patients served as the comparison group. Cases of HZ and related complications were ascertained from medical claims. Incidence rates (IR) and adjusted IR ratios were reported.

**Results:**

The study population consisted of 82,832 hematologic (HEM) and 944,777 solid cancer patients (SOLID). During follow-up, 9.2% of HEM and 6.3% of SOLID were diagnosed with HZ. The IR of HZ was significantly higher in HEM than SOLID (31.0 vs. 14.9 per 1,000 patient-years, p <0.01). The adjusted IR ratio vs. non-cancer elderly patients was 2.4 in HEM and 1.2 in SOLID. The proportion of patients with complications was higher in HEM than SOLID (17.8% vs. 15.8%, p <0.01). Age, gender, race, certain cancer therapies, and immunosuppression were HZ risk factors.

**Conclusions:**

Elderly cancer patients run a 1.2-2.4 times higher risk of developing HZ than those without cancer. The rates of HZ and HZ-related complications are significantly higher for hematologic than solid cancer patients.

**Electronic supplementary material:**

The online version of this article (doi:10.1186/s12879-015-0810-6) contains supplementary material, which is available to authorized users.

## Background

herpes zoster (HZ) is a vesicular rash associated with acute pain, caused by the varicella zoster virus (VZV) [1]. An estimated 1 million cases of HZ are reported annually in the US alone [2]. The risk of HZ increases when VZV-specific cell-mediated immunity declines, as a result of older age and/or immunosuppressive medical conditions and treatments [3,4].

There is sparse knowledge about the epidemiology of HZ in cancer patients, particularly among the elderly. Estimates from existing literature indicate the incidence of HZ is 2–4 times higher in cancer patients compared to the general population. The most recent literature on HZ in cancer patients covers commercially-insured patients diagnosed with cancer during 2001–2005 [5,6]. This study contributes to the body of literature by analyzing patients diagnosed with cancer during 1991–2007 and in the Medicare population.

The objectives of this study were to estimate the incidence of HZ, HZ-related pain, and HZ complications in elderly patients with selected hematologic and solid cancers, and to investigate risk factors for developing HZ in this population. Cancers were selected based on their prevalence as well as their known elevated risk of HZ.

## Methods

### Data source

This study utilized the Surveillance, Epidemiology, and End Results (SEER)-Medicare database, derived from a link between two large population-based data sources: the SEER cancer registries data, which cover approximately 28% of the US population [7] and contain information on cancer diagnoses, tumors, and mortality; and the Medicare enrollment and claims files, which cover the majority of US population aged ≥65 and contain charges and reimbursements for hospital, outpatient, physician, and home health claims. The link between the two databases was created by the US National Cancer Institute and the Centers for Medicare and Medicaid Services by matching individual identifiers from registries participating in the SEER program with identifiers contained in Medicare’s master enrollment file. The linked SEER-Medicare data include the records of approximately 93% of SEER cancer patients aged ≥65 [8]. The number of cancer registries covered by the SEER program has expanded over time, including during the period covered by our study.

The SEER program collects information about primary cancers such as cancer site, stage, histology, and first course of treatment for persons newly diagnosed with cancer who reside in one of the SEER geographic areas. The Medicare master enrollment file was used to determine which persons in the SEER data are Medicare beneficiaries. For people who are Medicare eligible, the SEER-Medicare data include claims for covered healthcare services, including hospital, physician, outpatient, home health, and hospice bills.

The analyses are based on Medicare claims during 1991–2009 of patients diagnosed with cancer during 1991–2007. The claims data include Medicare Part D pharmacy claims during 2007–2009. In addition, a cohort of non-cancer patients comprised of a random 5% sample of Medicare beneficiaries residing in the SEER registry areas is contained in the SEER-Medicare data and was included in our study for comparison. The New England Institutional Review Board approved this retrospective analysis of SEER-Medicare data as exempt from review due to the research involving the study of existing data (informed consent was waived).

### Study design

This was a retrospective, longitudinal, open cohort study. From the set of all Medicare-eligible patients in the SEER registry who were diagnosed with cancer from 1991–2007, those who were ≥65 years old at the time of cancer diagnosis, had ≥6 months of continuous insurance eligibility prior to cancer diagnosis, were covered by both Part A and Part B Medicare without Health Maintenance Organization enrollment, and had no evidence of cancer therapy or HZ diagnosis prior to cancer diagnosis were included in the main sample. Patients in the non-cancer comparison cohort were selected based on the same criteria.

The index date for cancer patients was defined as the date of cancer diagnosis. For the non-cancer cohort, an index date was needed in order to facilitate comparisons with the cancer cohorts. This index date for non-cancer patients was randomly assigned such that, following assignment, the mean and standard deviation of Medicare eligibility prior to the index date were the same as those for cancer patients. The observation period ran from the index date to the earlier of the end of data availability (December 31, 2009) or death.

### Definitions

An incident HZ case was defined as the first claim for an HZ episode (ICD-9 codes 053.X except 053.12 and 053.13) [9]. Both generalized and localized HZ cases were included. HZ-associated cutaneous, visceral, neurological, and ocular complications were identified by ICD-9 codes on claims within 90 days after any HZ claim in cases for which there was no claim for the complication prior to the date of first HZ diagnosis [2,10,11]. Given the time period and availability of data, the following criteria for HZ-related pain applied to the vast majority of patients: (i) two or more visits with an HZ diagnosis more than 28 days apart; or (ii) a diagnosis for HZ with other nervous system complications (ICD-9-CM 053.1x) or a diagnosis for neuralgia (ICD-9-CM 729.2) within 180 days of a visit for HZ. With the availability of Medicare Part D data beginning in 2007, a new prescription for a medication used to treat HZ-related pain was also required within 60 days of the HZ visits in the first criterion. A similar definition was adopted by Klompas, *et al*. to address the under-coding of post-herpetic neuralgia [12]. This definition accounts for the possibility that pain is underreported in the claims data by allowing for HZ-related pain without a pain claim (see Additional file 1: Table S1 for codes used to identify pain and pain interventions). We include in our estimates of the percentage of patients instances in which claims for HZ-related complications (see Additional file 1: Table S2 for the codes used to identify HZ-related complications) occur on the same claim as the initial HZ diagnosis since this is a relatively frequent occurrence in the data. In these instances, we cannot be certain that what we are observing are incident complications. Hence, the estimates of the percentage of patients developing each complication can be characterized as the prevalence of the complication within the 90-day period following HZ diagnosis.

### Statistical analysis

Demographic characteristics, observation time pre- and post-index date, and the Charlson Comorbidity Index (see Additional file 1: Table S3 for the description of the Charlson Comorbidity Index) [13-15] were reported. Cancer patients were stratified by cancer site and into cohorts based on cancer type (hematologic and solid). Frequencies and proportions (means and standard deviations) were reported for categorical variables (continuous variables). Differences between any cancer and non-cancer, hematologic and non-cancer, and solid and non-cancer patients were tested using chi-squared and t-tests for categorical and continuous variables, respectively.

The HZ incidence rate (IR) was estimated as the number of patients with incident HZ divided by person-years (PY) of observation censored at the earlier of first event or death. Confidence intervals (CI) were estimated using the exact Poisson confidence limit. Results were stratified by cancer site. A z-test was used to assess the difference between IRs of HZ for hematologic vs. solid cancers.

Adjusted incidence rate ratios (IRR) of HZ in cancer vs. non-cancer patients and 95% CIs were estimated by Poisson regressions using the covariates age at diagnosis, gender, HIV-positive, diabetic, race, and immunocompromising conditions/medications (e.g., rheumatoid arthritis, lupus erythematosus, medications inhibiting tumor necrosis factor – see Additional file 1: Table S3 for description of immunocompromising conditions/medications) to assess the incremental risk of HZ for cancer vs. non-cancer patients. Separate regressions were run on the samples of hematologic and solid cancer patients. Risk factors for developing HZ were assessed with IRRs estimated from Poisson regressions with the same set of covariates listed above as well as receipt of certain cancer therapies (hematologic cancer patients: autologous and allogeneic stem cell transplants; solid cancer patients: radiotherapy) and cancer stage at diagnosis (solid cancer patients only). Covariates whose estimated IRRs differed significantly from 1.0 were deemed to be risk factors for HZ. The proportions of patients with HZ at different time points during follow-up were also calculated. Receipt of chemotherapy, which was expected to be highly correlated with receipt of antiviral prophylaxis, could not be assessed as an independent HZ risk factor due to the lack of available pharmacy claims data prior to 2007.

Frequencies of events, observation time, proportions of HZ patients, and IRs for HZ-related complications were reported separately for the hematologic and solid cohorts. Events, observation time, and proportion of patients were based on complications diagnosed within 90 days of HZ diagnosis, and should thus be interpreted as the 90 day prevalence of complications. The IRs for HZ-related complications were calculated by Poisson regression with a log link. Patients with complications diagnosed on the same day as HZ are excluded from the regression due to lack of follow-up time. Reported estimates of 95% CIs for the IRs were estimated using the exact Poisson confidence limit. Frequencies of events and proportions of patients with HZ-related pain were reported by cancer site. Z-tests were used to assess the difference between the proportions of hematologic and solid cancer patients who developed HZ-related complications and pain.

All data analyses were performed using SAS release 9.2 or newer (SAS Institute, Inc., Cary, NC).

## Results

### Sample characteristics

**Figure 1:**
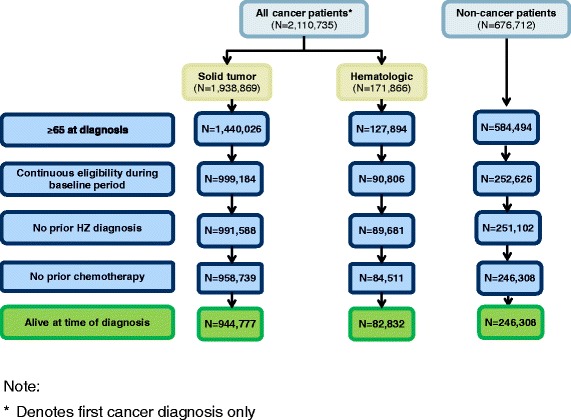
**Study population disposition.** The flow chart depicts how the selection criteria affect the number of patients remaining in the study sample. Each box represents the number of patients remaining in the sample at each step. From an initial sample of 2,110,735 cancer and 676,712 non-cancer patients, the final sample included 82,832 hematologic, 944,777 solid, and 246,308 non-cancer patients.

From an initial sample of 2,110,735 cancer and 676,712 non-cancer patients, the final sample included 82,832 hematologic, 944,777 solid, and 246,308 non-cancer patients (see Figure 1). Patient baseline characteristics are presented in Table 1. Significant differences existed between cancer and non-cancer patients in all characteristics considered. Due to the large sample size, not all statistically significant differences were meaningful. The average age at the index date was approximately 75–78 years across the cohorts. Compared with non-cancer patients, hematologic and solid cancer patients were slightly older on average (mean ages 77.5 and 76.1 vs. 74.7 years, p-values <0.0001). Most (62.7%) non-cancer patients were female, while hematologic cancer patients were roughly evenly divided across genders (51.0% female) and solid cancer patients tended to be male (55.1%). Most (84-89%) patients were White in race. Compared with non-cancer patients, larger proportions of hematologic and solid cancer patients were White (89.3% and 86.4%, respectively, vs. 84.1%), while smaller proportions were Asian/Pacific Islanders (2.2-2.3% vs. 3.2%) and North American Native/Alaska Native (0.2% vs. 0.4%). A smaller proportion of hematologic cancer patients were Black (4.7%) compared with solid cancer (7.9%) and non-cancer (7.6%) patients. Average follow-up times for hematologic and solid cancer patients were 37.8 and 52.1 months, respectively, and were significantly shorter compared to non-cancer patients (mean 85.3 months, p-values <0.0001).

**Table 1:** **Patient baseline characteristics**

	**Hematologic cancer patients** ^**1**^	**Solid cancer patients** ^**2**^	**Non-cancer patients**	**P-value hematologic cancer vs. non-cancer**	**P-value solid cancer vs. non-cancer**
Age at index date, mean ± SD^3^	77.5 ± 7.3	76.1 ± 6.9	74.7 ± 7.2	<0.0001	<0.0001
Gender, n (%)					
Male	40,516 (49.0%)	516,011 (55.1%)	91,940 (37.3%)	<0.0001	<0.0001
Female	42,232 (51.0%)	419,916 (44.9%)	154,368 (62.7%)	<0.0001	<0.0001
Race, n (%)					
White	73,895 (89.3%)	808,178 (86.4%)	207,161 (84.1%)	<0.0001	<0.0001
Black	3,923 (4.7%)	74,168 (7.9%)	18,760 (7.6%)	<0.0001	<0.0001
Asian/Pacific Islander	1,800 (2.2%)	21,116 (2.3%)	7,811 (3.2%)	<0.0001	<0.0001
Hispanic	1,203 (1.5%)	12,237 (1.3%)	5,896 (2.4%)	<0.0001	<0.0001
North American Native/Alaska Native	155 (0.2%)	1,952 (0.2%)	901 (0.4%)	<0.0001	<0.0001
Other	1,489 (1.8%)	15,674 (1.7%)	5,013 (2.0%)	<0.0001	<0.0001
Months before the index date, mean ± SD^3^	110.9 ± 64.8	101.4 ± 62.4	85.6 ± 47.6	<0.0001	<0.0001
Months after the index date, mean ± SD^3^	37.8 ± 41.3	52.1 ± 48.8	85.3 ± 62.6	<0.0001	<0.0001
Charlson Comorbidity Index, mean ± SD^3^	0.7 ± 1.1	0.7 ± 1.1	0.5 ± 0.9	<0.0001	<0.0001

Notes:

^1^Hematologic cancers include chronic lymphocytic leukemia, acute lymphocytic or acute myeloid leukemia, other leukemia, Hodgkin’s lymphoma, and non-Hodgkin’s lymphoma.

^2^Solid cancers include bladder cancer, breast cancer, colorectal cancer, head and neck cancer, lung cancer, melanoma, ovarian cancer, prostate cancer, and soft tissue sarcoma.

^3^SD = standard deviation

### Incidence rates of herpes zoster

Results on the IR of HZ are presented in Table 2. Across the cancer types considered, the IR of HZ was lowest for patients with prostate cancer and highest for patients with Hodgkin’s Lymphoma (12.3 and 47.8 per 1,000 PY, respectively). The IR of HZ was significantly higher in the hematologic vs. solid cancer cohort (31.0 vs. 14.9 per 1,000 PY, p < 0.01). Both hematologic and solid cancer patients were more likely to develop HZ compared to non-cancer patients (adjusted IRRs of 2.36 and 1.19, p-values < 0.001).

**Table 2:** **Incidence of herpes zoster in cancer patients**

	**Patients**	**First events**	**Person-years of observation**	**IR Per 1,000 person-years (95% CI)** ^**1**^	**Adjusted IRR vs. non-cancer patients (95% CI)** ^**1**^	**P-value**
**All Cancers**	**1,027,609**	**66,222**	**4,161,133**	**15.9 (15.8-16.0)**	**1.27 (1.25-1.29)**	**<0.001**
**Hematologic Cancers** ^**2**^	**82,832**	**7,574**	**244,187**	**31.0 (30.3-31.7)**	**2.36 (2.30-2.42)**	**<0.001**
Chronic Lymphocytic Leukemia	14,035	1,754	56,875	30.8 (29.4-32.3)	2.37 (2.26-2.49)	<0.001
Acute Lymphocytic or Acute Myeloid Leukemia	9,614	257	7,125	35.9 (31.8-40.6)	2.75 (2.43-3.11)	<0.001
Other Leukemia	7,803	391	15,685	24.9 (22.5-27.5)	1.90 (1.72-2.11)	<0.001
Hodgkin’s Lymphoma	2,147	283	5,923	47.8 (42.5-53.7)	3.56 (3.17-4.01)	<0.001
Non-Hodgkin’s Lymphoma	49,233	4,891	158,773	30.7 (29.9-31.6)	2.31 (2.24-2.39)	<0.001
**Solid Cancers** ^**2**^	**944,777**	**58,670**	**3,921,307**	**14.9 (14.8-15.1)**	**1.19 (1.17-1.21)**	**<0.001**
Bladder Cancer	67,811	4,140	293,432	14.1 (13.7-14.5)	1.11 (1.08-1.16)	<0.001
Breast Cancer	173,635	15,819	963,395	16.4 (16.2-16.7)	1.17 (1.14-1.19)	<0.001
Colorectal Cancer	129,761	7,354	504,238	14.6 (14.2-14.9)	1.11 (1.08-1.14)	<0.001
Head and Neck Cancer	48,876	2,530	173,559	14.6 (14.0-15.2)	1.14 (1.09-1.19)	<0.001
Lung Cancer	200,734	6,376	287,656	22.1 (21.6-22.7)	1.61 (1.56-1.66)	<0.001
Melanoma	52,026	3,961	268,256	14.8 (14.3-15.2)	1.15 (1.11-1.19)	<0.001
Ovarian Cancer	18,715	844	48,762	17.3 (16.2-18.5)	1.21 (1.13-1.30)	<0.001
Prostate Cancer	247,939	17,371	1,406,361	12.3 (12.2-12.5)	1.12 (1.09-1.15)	<0.001
Soft Tissue Sarcoma	5,280	275	18,206	15.1 (13.4-16.9)	1.16 (1.03-1.31)	0.016

Notes:

^1^IR = incidence rate; IRR = IR ratio; CI = confidence interval.

^2^P-value of difference in IR of HZ for hematologic vs. solid cancer <0.01.

**Figure 2:**
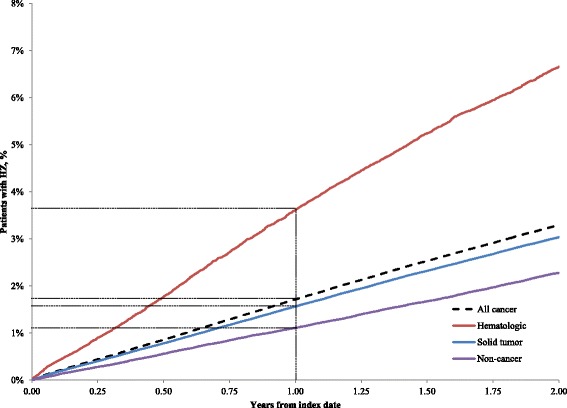
**Proportions of patients developing herpes zoster after cancer diagnosis.** The proportions of patients developing HZ by time since index date are plotted. Cancer patients in this analysis were required to have survived for at least 2 years after cancer diagnosis. The proportion of patients being diagnosed with HZ was plotted for the overall, hematologic, solid tumor, and non-cancer cohorts. Dashed lines indicate the percentage of each cohort with HZ at 1 year since the index date. Approximately 3.75% and 1.50% of patients in the hematologic and solid cancer cohorts developed HZ within 1 year of cancer diagnosis, compared with 1.10% of non-cancer patients. The proportions of hematologic and solid cancer patients who developed HZ within 2 years of cancer diagnosis were 6.6% and 3.0%, respectively.

The proportions of patients developing HZ by time since index date are plotted in Figure 2 (cancer patients in this analysis were required to have survived for at least 2 years after cancer diagnosis). Approximately 3.75% and 1.50% of patients in the hematologic and solid cancer cohorts developed HZ within 1 year of cancer diagnosis, compared with 1.10% of non-cancer patients. The proportions of hematologic and solid cancer patients who developed HZ within 2 years of cancer diagnosis were 6.6% and 3.0%, respectively. Overall, 9.2% of hematologic and 6.3% of solid cancer patients were diagnosed with HZ during follow-up. Among patients who developed HZ, the median time of HZ onset following cancer diagnosis was 34.0 months (24.8 and 35.4 months for hematologic and solid tumor patients, respectively).

### herpes zoster risk factors

Results from the analysis of risk factor for HZ are presented in Table 3. Age, gender, race, and immunocompromising conditions and/or medications were statistically significant risk factors for HZ. Conditional on cancer status, estimates from the sample that included hematologic cancer patients indicate that patients 75 to 85 years old ran a higher risk of developing HZ compared with patients ≥85 years (IRR = 1.12, p < 0.001). Males were at lower risk of developing HZ than females (IRR = 0.83, p < 0.001). Black patients were less likely to develop HZ than white patients (IRR = 0.64, p < 0.001). Patients who had immunocompromising conditions other than HIV ran a higher risk of developing HZ (IRR = 1.25, p < 0.001). Finally, receipt of autologous or allogeneic stem cell transplants was associated with a higher risk of developing HZ (IRRs of 2.07, p < 0.001; and 1.92, p = 0.024, respectively). Results from the sample that included solid cancer patients were largely similar. Radiotherapy recipients ran a slightly lower risk of developing HZ compared to solid cancer patients who did not receive radiotherapy (IRR = 0.94, p < 0.001) while patients with stage III/IV cancer at diagnosis ran a higher risk of developing HZ (IRR = 1.20, p < 0.001).

**Table 3:** **Risk factors for herpes zoster**

**Variable**	**Hematologic cancers**			**Solid cancers**		
	**(n = 82,832)**			**(n = 944,777)**		
	**Incidence rate ratio**	**95% CI** ^**1**^	**P-value**	**Incidence rate ratio**	**95% CI** ^**1**^	**P-value**
Age at diagnosis (ref = 85+ years old)						
65 to 75 years old	1.05	(1.00 - 1.10)	0.067	0.97	(0.94 - 1.00)	0.025
75 to 85 years old	1.12	(1.07 - 1.18)	<0.001	1.04	(1.01 - 1.07)	0.014
Male (ref = female)	0.83	(0.81 - 0.85)	<0.001	0.80	(0.78 - 0.81)	<0.001
Race (ref = white)						
Black	0.64	(0.50 - 0.81)	<0.001	0.60	(0.51 - 0.70)	<0.001
Asian/Pacific Islander	1.25	(0.98 - 1.60)	0.074	1.28	(1.08 - 1.51)	0.003
North American Native/Alaska Native	1.06	(0.77 - 1.46)	0.730	0.92	(0.74 - 1.16)	0.488
Other or Unknown	1.11	(0.87 - 1.43)	0.398	1.16	(0.98 - 1.37)	0.089
Diabetes	1.02	(0.98 - 1.07)	0.344	0.99	(0.96 - 1.01)	0.225
HIV-positive (ref = HIV-negative)	0.82	(0.41 - 1.65)	0.587	1.16	(0.78 - 1.72)	0.451
Other immunocompromising conditions and/or medications^2^	1.25	(1.20 - 1.30)	<0.001	1.26	(1.23 - 1.28)	<0.001
Cancer patient	2.35	(2.29 - 2.42)	<0.001	1.18	(1.15 - 1.21)	<0.001
Receipt of autologous stem cell transplant	2.07	(1.49 - 2.89)	<0.001	N/A		
Receipt of allogeneic stem cell transplant	1.92	(1.09 - 3.38)	0.024			
Receipt of radiotherapy	N/A			0.94	(0.93 - 0.96)	<0.001
Stage I/II at diagnosis				1.01	(0.99 - 1.03)	0.269
Stage III/IV at diagnosis				1.20	(1.16 - 1.23)	<0.001

Notes:

^1^CI = confidence interval.

^2^See Additional file 1: Table S3 for the definition of other immunocompromising conditions and/or medications.

### herpes zoster-related complications and pain

Results on the incidence of HZ-related complications in hematologic and solid cancer patients are presented in Table 4. Among hematologic cancer patients, the range of IRs across the different types of complications considered was wide (0.0 for deafness, other neurological complications, and panopthalmitis to 132.5 for postherpetic polyneuropathy and postherpetic trigeminal neuralgia). 17.8% of hematologic cancer patients developed some type of complication (IR = 530.0 per 1,000 PY). Ophthalmic complications were the most prevalent (11.3% of patients; IR = 247.3 per 1,000 PY) compared with the other types of complications considered aside from HZ-related pain. Similar proportions of hematologic and solid cancer patients developed HZ-related complications across the major categories of complications considered, although a significantly larger share of hematologic cancer patients developed complications (17.8% vs. 15.8% of patients, p < 0.01).

**Table 4:** **Incidence of HZ complications in hematologic and solid cancer patients**

**Outcome measure**	**Hematologic cancer patients (n = 7,574)**				**Solid cancer patients (n = 58,670)**			
	**First events**	**Person-years of observation**	**% with event**	**Incidence rate per 1000 person-years (95% CI)** ^**1,2**^	**First events**	**Person-years of observation**	**% with event**	**Incidence rate per 1000 person-years (95% CI)** ^**1,2**^
**Any Complication** ^**3,4**^	**1,348**	**1,421**	**17.8**	**530.0 (493.5-569.3)**	**9,253**	**11,483**	**15.8**	**398.3 (386.9-410.0)**
**Cutaneous Complications**	**291**	**1,652**	**3.8**	**122.8 (107.1-141.0)**	**1,462**	**13,296**	**2.5**	**73.6 (69.1-78.3)**
Cellulitis	289	1,653	3.8	122.2 (106.5-140.3)	1,456	13,297	2.5	73.2 (68.7-77.9)
Zoster gangrenosum	<11	1,776	<0.2	0.6 (0.1-4.0)	<11	13,973	<0.1	0.4 (0.1-0.9)
**Visceral Complications**	**<11**	**1,775**	**<0.2**	**3.4 (1.5-7.5)**	**<11**	**13,974**	<**0.1**	**0.2 (0.1-0.7)**
Pneumonitis	<11	1,775	<0.2	3.4 (1.5-7.5)	<11	13,974	<0.1	0.2 (0.1-0.7)
**Neurological Complications**	**339**	**1,716**	**4.5**	**174.8 (156.1-195.7)**	**1,860**	**13,633**	**3.2**	**121.7 (116.0-127.7)**
Aseptic meningitis	<11	1,775	<0.2	4.5 (2.3-9.0)	30	13,969	0.1	1.5 (1.0-2.3)
Cranial nerve palsies	42	1,766	0.6	18.1 (12.8-25.6)	223	13,918	0.4	11.9 (10.2-13.8)
Deafness	<11	1,777	<0.2	0.0 (0.0-0.0)	<11	13,974	<0.1	0.1 (0.0-0.6)
Diaphragmatic paralysis	<11	1,776	<0.2	2.8 (1.2-6.8)	52	13,964	0.1	3.2 (2.3-4.2)
Encephalitis	26	1,772	0.3	13.0 (8.6-19.5)	106	13,953	0.2	6.5 (5.3-8.0)
Motor neuropathy	<11	1,776	<0.2	0.6 (0.1-4.0)	17	13,971	0.0	1.2 (0.8-2.0)
Postherpetic polyneuropathy and postherpetic trigeminal neuralgia	252	1,735	3.3	132.5 (116.5-150.8)	1,393	13,739	2.4	93.7 (88.8-99.0)
Sensory loss	<11	1,775	<0.2	4.5 (2.3-9.0)	66	13,959	0.1	4.7 (3.7-6.0)
Transverse myelitis	<11	1,776	<0.2	1.1 (0.3-4.5)	16	13,972	0.0	1.1 (0.7-1.9)
Other complications	<11	1,777	<0.2	0.0 (0.0-0.0)	<11	13,975	<0.1	0.0 (0.0-0.0)
**Ophthalmic Complications**	**853**	**1,565**	**11.3**	**247.3 (223.8-273.2)**	**6,689**	**12,276**	**11.4**	**218.5 (210.4-226.9)**
Dermatitis of eyelid	337	1,700	4.4	94.1 (80.6-109.9)	2,626	13,363	4.5	79.8 (75.1-84.7)
Iridocyclitis/Uveitis/ Chorioretinitis/Retinitis	84	1,759	1.1	31.3 (24.0-40.7)	624	13,836	1.1	31.2 (28.4-34.3)
Keratoconjunctivitis	289	1,711	3.8	75.4 (63.4-89.6)	2,058	13,499	3.5	60.3 (56.3-64.6)
Keratitis	95	1,752	1.3	49.1 (39.7-60.6)	641	13,786	1.1	39.0 (35.8-42.4)
Mydriasis	<11	1,777	<0.2	0.6 (0.1-4.0)	<11	13,975	<0.1	0.1 (0.0-0.5)
Panopthalmitis	<11	1,777	<0.2	0.0 (0.0-0.0)	<11	13,975	<0.1	0.0 (0.0-0.0)
Ptosis	16	1,771	0.2	9.0 (5.5-14.7)	184	13,928	0.3	12.3 (10.6-14.3)
Scleritis	111	1,735	1.5	54.2 (44.3-66.3)	758	13,632	1.3	43.4 (40.1-47.1)
Visual impairment	33	1,768	0.4	15.8 (10.9-22.9)	204	13,915	0.3	12.2 (10.5-14.2)
**Other complications**	**209**	**1,731**	**2.8**	**56.6 (46.4-69.0)**	**1,687**	**13,592**	**2.9**	**47.3 (43.8-51.1)**

Notes:

^1^First events, person-years of observation, and % with event based on complications diagnosed within 90 days of HZ diagnosis and should thus be interpreted as the 90-day prevalence of complications. Incidence rates are estimated by Poisson regression with a log link. Patients with complications diagnosed on the same day as HZ are excluded from the regression due to lack of follow-up time. Thus, dividing the number of first events by the person-years of observation will not match the incidence rate estimates.

^2^CI = confidence interval.

^3^The p-value of the difference in % with any complication for hematologic vs. solid cancer was <0.01.

^4^See Additional file 1: Table S2 for ICD-9 codes used to define complications.

Results on the incidence of HZ-related pain are presented in Table 5. The proportions of hematologic and solid cancer patients who developed HZ-related pain were 47.7% and 41.0%, respectively. Significantly higher proportions of hematologic cancer patients developed HZ-related pain compared to solid cancer patients (p-values < 0.01).

**Table 5:** **Incidence of herpes zoster-related pain**

**Outcome Measure**	**First events**	**Population** ^**1**^	**% with herpes zoster-related pain**
**All Cancer**	**27,664**	**66,222**	**41.8**
**Hematologic Cancers** ^**2**^	**3,611**	**7,574**	**47.7**
Chronic Lymphocytic Leukemia	914	1,754	52.1
Acute Lymphocytic or Acute Myeloid Leukemia	79	257	30.7
Other Leukemia	161	391	41.2
Hodgkin’s Lymphoma	133	283	47.0
Non-Hodgkin’s Lymphoma	2,324	4,891	47.5
**Solid Cancers** ^**2**^	**24,070**	**58,670**	**41.0**
Bladder Cancer	1,716	4,140	41.4
Breast Cancer	6,822	15,819	43.1
Colorectal Cancer	3,010	7,354	40.9
Head and Neck Cancer	1,030	2,530	40.7
Lung Cancer	2,495	6,376	39.1
Melanoma	1,666	3,961	42.1
Ovarian Cancer	343	844	40.6
Prostate Cancer	6,847	17,371	39.4
Soft Tissue Sarcoma	141	275	51.3

Notes:

^1^Patients were observed following the incident diagnosis of herpes zoster.

^2^P-values of differences in % of patients with herpes zoster-related pain for hematologic vs. solid cancer <0.01.

## Discussion

This study of elderly patients was undertaken to describe the characteristics of patients with and without cancer, provide a comprehensive analysis of the incidence and onset of HZ, HZ-related pain, and HZ complications in elderly cancer patients, and assess risk factors for developing HZ. The SEER-Medicare database facilitated the use of large samples of cancer patients, as well as a sample of non-cancer patients for comparison purposes.

This study found significantly higher HZ incidence rates in cancer vs. non-cancer patients. Specifically, elderly cancer patients ran a 1.2-2.4 times higher risk of developing HZ than those without cancer. Incidence rates of HZ were lowest among patients with prostate cancer and highest among patients with Hodgkin’s Lymphoma. Hematologic cancer patients tended to have the highest HZ incidence rates and develop HZ more quickly following cancer diagnosis. These findings are consistent with the previous findings of Habel, *et al*. and Rusthoven, *et al.* [6,16]. In particular, the HZ incidence rates reported by Habel, *et al*. in a study of a commercially-insured cancer population (31 and 12 per 1,000 PY for hematologic and solid cancer patients, respectively) are very similar to those found here [6]. In addition, the proportions of hematologic and solid cancer patients developing HZ within 2 years of cancer diagnosis found in this study (6.6% and 3.0%, respectively) are comparable to those reported by Habel *et al*. (6% and 2%, respectively). Hematologic cancer patients also developed HZ-related complications and pain more frequently than solid cancer patients. The proportion of hematologic cancer patients with HZ developing neurologic complications (4.5%) was comparable to, and developing cutaneous and ophthalmic complications differed from, those reported elsewhere in the literature [6,17]. In particular, the proportion of hematologic cancer patients with HZ that developed ophthalmic complications found in this study (11.3%) is substantially larger than that reported by Habel *et al*. (3.6%), as well as that reported by Yawn *et al*. in a study of HZ in non-cancer patients in Olmsted County, MN based on medical record review (4.1%) [6,17].

Risk factors for developing HZ included age, gender, race, immunosuppressive conditions, and certain cancer therapies. Males and black patients (relative to white patients) had a lower risk for developing HZ, consistent with data reported by Opstelten *et al*. [18] (gender risk factors) and Schmader *et al*. [19,20] (race risk factors). Hematologic or solid cancer patients with immunocompromising conditions ran a higher risk of developing HZ, as did hematologic cancer patients who received stem cell transplants (despite the routine use of HZ prophylaxis post-transplant). Additionally, solid cancer patients with a stage III/IV cancer were at a higher risk for developing HZ. Cancer patients aged 75 to 85 years old had a higher risk of developing HZ than patients 85 years and older. This somewhat counterintuitive result may reflect different approaches (e.g., more aggressive chemotherapies, inducing greater immune suppression) used to treat elderly cancer patients aged 75 to 85 for cancer and other illnesses compared with approaches used to treat elderly cancer patients 85 years and older. These different treatment approaches may lead to different HZ risks. Habel, *et al*. reports a similar decreased risk of HZ for cancer patients 80 years and older compared to those younger than 80 years, but only among patients with hematologic cancers [6].

There is a wide range of estimates of post-herpetic neuralgia incidence ranging from <1% to 70% previously reported in the literature, with this study reporting proportions of patients with HZ-related pain (41.0% and 47.7% for solid and hematologic cancer patients, respectively) on the higher end [21-23]. This higher estimate may be a result of a possible lack of specificity of the claims data definition employed on the cancer population. Cancer patients may have other painful conditions associated with their oncological therapy that may be getting attributed to HZ-related pain.

This study has several notable limitations. As with all claims database analyses, ICD-9 codes were used to identify diagnoses; these codes may not reflect confirmed clinical diagnoses and lack information to assess severity of illness. Medical services obtained outside of a patient’s plan are not captured in claims data, so HZ incidence in this study may be underestimated. The analysis of HZ-related pain may not reflect the true incidence of HZ-related pain in the studied populations. Specifically, the definition may cause our study to overstate the incidence of HZ-related pain among cancer patients compared with non-cancer patients by classifying general cancer-related pain as HZ-related pain, particularly since pain may be more likely to be diagnosed in cancer patients (who likely receive regular evaluations and treatment) compared with non-cancer patients. No validation of codes and algorithms for identifying HZ and its complications was performed. However, a study that performed validation with comparison to electronic medical records found high positive predictive value (PPV) for identifying HZ and its complications (78%-93%) [12]. The SEER-Medicare data extract used in this study did not include patients with multiple myeloma. Receipt of chemotherapy was not analyzed as an HZ risk factor due to the lack of available pharmacy claims data prior to 2007. Finally, some of the steroids defined as immunosuppressive treatments could be part of a cancer treatment protocol.

## Conclusions

The results of this study indicate that elderly cancer patients face an increased risk of HZ compared to non-cancer patients. Elderly patients with hematologic cancers are particularly vulnerable to HZ and related complications and pain. Gender, race, and certain immunocompromising conditions and certain cancer therapies are risk factors for HZ. Further research on HZ in cancer patients is needed within the context of non-Medicare populations as well as patients with multiple myeloma.

### Ethics statement

The New England Institutional Review Board approved this retrospective analysis of Surveillance Epidemiology and End Results Medicare data for Medicare beneficiaries. This research was determined to be exempt from NEIRB review as it involved study of existing data. Informed consent was waived since the study does not involve intervention or interactions with individuals, only registry and claims data are used. The SEER-Medicare contact at the National Cancer Institute reviewed the manuscript and verified that there are no confidentially issues with the presented data.

## Additional file

Additional file 1: Table S1. Codes to Identify Pain and Pain Interventions. **Table S2.** ICD-9 Codes to Identify HZ Complications. **Table S3.** Additional Variable Descriptions.

## Abbreviations

HZ: herpes zoster
VZV: Varicella Zoster Virus
SEER: Surveillance, Epidemiology, and End Results
IR: Incidence rate
PY: Person-years
CI: Confidence intervals
IRR: Incidence rate ratios

## References

[1] Arvin AM, Gershon A (2000). Varicella-Zoster Virus – Virology and Clinical Management.

[2] Harpaz R, Ortega-Sanchez IR, Seward JF (2008). Advisory Committee on Immunization Practices (ACIP) Centers for Disease Control and Prevention (CDC). Prevention of herpes zoster: recommendations of the Advisory Committee on Immunization Practices (ACIP). MMWR Recomm Rep.

[3] Dworkin RH, Johnson RW, Breuer J, Gnann JW, Levin MJ, Backonja M (2007). Recommendations for the management of herpes zoster. Clin Infect Dis.

[4] Cohen J (2013). Herpes Zoster. N Engl J Med.

[5] Habel LA, Ray GT, Horberg M, Yawn B, Castillo A, Li Y (2009). The epidemiology of herpes zoster in patients with invasive cancer. J Clin Oncol.

[6] Habel LA, Ray GT, Silverberg MJ, Horberg MA, Yawn BP, Castillo AL (2013). The epidemiology of herpes zoster in patients with newly diagnosed cancer. Cancer Epidemiol Biomarkers Prev.

[7] National Cancer Institute (NCI): SEER. [http://seer.cancer.gov/about/overview.html] Accessed Nov. 7, 2013.

[8] NCI: SEER-Medicare: How the SEER & Medicare Data are Linked. [http://healthservices.cancer.gov/seermedicare/overview/linked.html] Accessed Nov. 7, 2013.

[9] Insinga RP, Itzler RF, Pellissier JM, Saddier P, Nikas AA (2005). The incidence of herpes zoster in a United States administrative database. J Gen Intern Med.

[10] Volpi A (2007). Severe complications of herpes zoster. Herpes.

[11] Weaver BA (2007). The burden of herpes zoster and postherpetic neuralgia in the United States. J Am Osteopath Assoc.

[12] Klompas M, Kulldorff M, Vilk Y, Bialek SR, Harpaz R (2011). Herpes Zoster and Postherpetic Neuralgia Surveillance using structured electronic data. Mayo Clin Proc.

[13] Charlson ME, Pompei P, Ales KL, MacKenzie CR (1987). A new method of classifying prognostic comorbidity in longitudinal studies: development and validation. J Chronic Dis.

[14] Deyo RA, Cherkin DC, Ciol MA (1992). Adapting a clinical comorbidity index for use with ICD-9-CM administrative databases. J Clin Epidemiol.

[15] Romano PS, Roos LL, Jollis JG (1993). Adapting a clinical comorbidity index for use with ICD-9-CM administrative data: differing perspectives. J Clin Epidemiol.

[16] Rusthoven JJ, Ahlgren P, Elhakim T, Pinfold P, Reid J, Stewart L (1988). Varicella-zoster infection in adult cancer patients: a population study. Arch Intern Med.

[17] Yawn BP, Wollan P, St. Sauver J (2011). Comparing shingles incidence and complication rates from medical record review and administrative database estimates: How close are they?. Am J Epidemiol.

[18] Opstelten W, Van Essen GA, Schellevis F, Verheij TJ, Moons K (2006). Gender as an independent risk factor for herpes zoster: a population-based prospective. Ann Epidemiol.

[19] Schmader K, George LK, Burchett BM, Pieper CF (1995). Racial differences in the occurrence of herpes zoster. J Infect Dis.

[20] Schmader K, George LK, Burchett BM, Hamilton JD, Pieper CF (1998). Race and stress in the incidence of herpes zoster in older adults. J Am Geriatr Soc.

[21] Gnann JW, Whitley RJ (2002). Clinical practice. Herpes zoster. N Engl J Med.

[22] Opstelten W, Mauritz JW, de Wit NJ, van Wijck AJ, Stalman WA, van Essen GA (2002). Herpes zoster and postherpetic neuralgia: incidence and risk indicators using a general practice research database. Fam Pract.

[23] Yawn BP, Saddier P, Wollan PC, St Sauver JL, Kurland MJ, Sy LS (2007). A population-based study of the incidence and complication rates of herpes zoster before zoster vaccine introduction. Mayo Clin Proc.

