# Supplementary material for: The epidemiology of herpes zoster and its complications in Medicare cancer patients
Source: BMC Infect Dis. 2015 Feb 27;15:106. doi: 10.1186/s12879-015-0810-6 (PMC4352235; doi:10.1186/s12879-015-0810-6)
Supplement: Additional file 1: Table S1. — Codes to Identify Pain and Pain Interventions. Table S2. ICD-9 Codes to Identify HZ Complications. Table S3. Additional Variable Descriptions. [file 12879_2015_810_MOESM1_ESM.docx]

## Additional files

### *Additional files - Table S1: Codes to Identify Pain and Pain Interventions*

| **ICD-9 Codes to Identify Pain** | | |
| --- | --- | --- |
| **Medical Term** | **Code(s)** | **Description** |
| Pain, not elsewhere classified | 338 | Pain, not elsewhere classified |
|  | 338.1 | Acute pain |
|  | 338.19 | Other acute pain |
|  | 338.2 | Chronic pain |
|  | 338.29 | Other chronic pain |
| Rheumatism, excluding the back | 729.2 | Neuralgia, neuritis, and radiculitis, unspecified |
| General pain | 780.96 | Generalized pain |
| **CPT Codes and ICD-9 Procedure  Codes to Identify Pain Intervention Procedures** | | |
| **Intervention** | **CPT Codes** | **ICD-9 Procedure Codes** |
| Acupuncture | 97801, 97811, 97813, 97814 | 99.82 |
| Introduction of anesthetic agent, nerve block | 64400-64530 | 04.8-04.89 or 05.31 |
| Neurostimulators | 63650, 63655, 63685, 64550-64595 | 03.93, 04.92, or 86.94-86.96 |
| Nerve destruction by neurolytic agent | 64600-64680 | 03.8x or 04.2x |
| **Pain Medications** | | |
| Non-opioid analgesics | Opioid analgesics | Anticonvulsants |
| Tricyclic antidepressants | Other antidepressants | Baclofen |
| Capsaicin cream | Clonidine | Diclofenac cream |
| Gabapentin | Lidocaine/Lidocaine patch | Mexiletine |
| Pamidronate | Prilocaine cream | Tizanidine |

### *Additional files - Table S2: ICD-9 Codes to Identify HZ Complications*

| **Complication** | **ICD-9 Codes** |
| --- | --- |
| **Cutaneous Complications** | |
| Cellulitis | 681.x, 682.x, 528.3, 528.5 |
| Zoster gangrenosum | 686.01 |
| **Visceral Complications** | |
| Pneumonitis | 052.1 |
| **Neurological Complications** | |
| Aseptic meningitis | 047.9 |
| Cranial nerve palsies | 352.6, 351.0 |
| Deafness | 389.17 |
| Diaphragmatic paralysis | 519.4 |
| Encephalitis | 323.x |
| Motor neuropathy: | 357.82 |
| Sensory loss | 389.11, 389.17 |
| Transverse myelitis | 341.x, 323.82 |
| HZ with other nervous system complications | 053.10-053.14, 053.19 |
| **Ophthalmic Complications** | |
| Dermatitis of eyelid | 053.20 |
| Iridocyclitis/uveitis/choriorentinitis/retinitis | 053.22, 364.3, 363.20 |
| Keratoconjunctivitis | 053.21 |
| Keratitis | 370.x |
| Mydriasis: | 379.43 |
| Panopthalmitis | 360.02 |
| Ptosis | 374.3 |
| Scleritis | 379.0 |
| Visual impairment | 369.x |
| Other ophthalmic complications | 053.29 |

### *Additional files - Table S3: Additional Variable Descriptions*

| **Variable** | **Description** |
| --- | --- |
| Charlson Comorbidity Index | Predicts the mortality for a patient who may have a range of comorbid conditions. Each condition is assigned with a score depending on the risk of dying associated with this condition. The conditions considered include: AIDS, metastatic solid tumor, liver and renal disease, malignancy, hemiplegia or paraplegia, diabetes mellitus, ulcer disease, rheumatologic disease, chronic pulmonary disease, dementia, cerebrovascular and peripheral vascular disease, congestive heart failure, and myocardial infarction. The score ranges from 0-37. An increase of one point is, for example, associated with diabetes without end-organ damage [14]. |
| Other Immunocompromising Conditions and/or Medications | Rheumatoid arthritis, lupus erythemotosus, Sjögren’s syndrome, polymyalgia rheumatic, chronic pulmonary disease, chronic kidney disease, scleroderma, and inflammatory bowel disease; heart, lung, cornea, liver, pancreas, and kidney transplant; and use of anti-tumor necrosis factors, other disease-modifying antirheumatic drugs and biologics, and any corticosteroid. |
